# Abnormal Cervical Vestibular-Evoked Myogenic Potentials Predict Evolution of Isolated Recurrent Vertigo into Meniere’s Disease

**DOI:** 10.3389/fneur.2017.00463

**Published:** 2017-09-04

**Authors:** Sun-Uk Lee, Hyo-Jung Kim, Jeong-Yoon Choi, Ja-Won Koo, Ji-Soo Kim

**Affiliations:** ^1^Department of Neurology, Seoul National University College of Medicine, Seoul National University Bundang Hospital, Seongnam, South Korea; ^2^Research Administration Team, Seoul National University Bundang Hospital, Seongnam, South Korea; ^3^Department of Otolaryngology, Seoul National University College of Medicine, Seoul National University Bundang Hospital, Seongnam, South Korea

**Keywords:** Meniere’s disease, vertigo, nystagmus, hearing loss, tinnitus, vestibular-evoked myogenic potentials

## Abstract

**Introduction:**

Vestibular-evoked myogenic potentials (VEMPs) can be abnormal in patients with idiopathic recurrent spontaneous vertigo. We aimed to determine whether abnormal cervical vestibular-evoked myogenic potentials (cVEMPs) can predict evolution of isolated recurrent vertigo into Meniere’s disease (MD).

**Methods:**

We had followed up 146 patients with isolated recurrent vertigo and an evaluation of cVEMPs for 0–142 months [median = 6, interquartile range (IQR) = 0–29] at the Dizziness Clinic of Seoul National University Bundang Hospital from June 2003 to May 2014. We defined the variables associated with a progression into MD and calculated cumulative progression rates.

**Results:**

Among the 94 patients with recurrent vertigo and abnormal cVEMPs, 18 (18/94, 19%) showed an evolution into MD while only 2 of the 50 (4%) patients with normal cVEMPs evolved into MD during the follow-up (*p* = 0.01). The interval between onset of vertigo and development of cochlear symptoms ranged from 1 month to 13.6 years (median = 3 years, IQR = 0.5–4.5 years). Overall, pure tone audiometry (PTA) threshold at 0.25 kHz [hazard ratio (HR) = 1.1, 95% confidence interval (CI) = 1.0–1.2] and abnormalities of cVEMPs (HR = 5.6, 95% CI = 1.3–25.5) were found to be significantly associated with a later conversion into MD. The cumulative progression rate was 12% (95% CI = 5–18) at 1 year, 18% (8–26) at 2 years, and 22% (11–32) at 3 years.

**Conclusion:**

Abnormal cVEMPs may be an indicator for evolution of isolated recurrent vertigo into MD. Patients with isolated recurrent vertigo may be better managed conforming to MD when cVEMPs are abnormal.

## Introduction

Meniere’s disease (MD) is characterized by recurrent spontaneous vertigo and fluctuating aural symptoms including tinnitus, ear fullness, and hearing loss (1). Current criteria require both vestibular and cochlear manifestations to diagnose MD (1). However, the initial developments of vestibular and cochlear symptoms may be separated in time in patients with MD (2). Thus, the vestibular symptoms may precede cochlear findings or *vice versa*. Indeed, about 20% of patients with recurrent vertigo eventually progress to typical MD (3). Previous diagnostic criteria for MD also included sub-categories on these conditions, so-called “vestibular or cochlear MD,” to embrace these patients (1, 2, 4). However, diagnosis of isolated recurrent vertigo remains a challenge since it may occur in various underlying disorders or may evolve into other disorders causing recurrent vertigo. Thus, it would be important to predict evolution of isolated recurrent vertigo into more severe form of disorders causing recurrent vertigo.

Introduction of vestibular-evoked myogenic potentials (VEMPs) has enabled us to evaluate the function of the otolithic organs, the saccule and utricle, more easily. Cervical VEMPs (cVEMPs) in response to air-conducted sounds are known to reflect the uncrossed inhibitory potentials originating from the saccule, while ocular VEMPs (oVEMPs) induced by bone-conducted vibration reflect the crossed excitatory potentials originating primarily from the utricle.

Given that the endolymphatic hydrops (EH) mostly starts from the cochlea, and then involves the saccule, which is the vestibular organ closest to the cochlea (5), VEMPs, especially the cVEMPs reflecting the function of the saccule, may be impaired in patients with MD manifesting with isolated recurrent vertigo. The aim of this study was to determine that abnormal cVEMPs and oVEMPs are indeed a predictor for evolution into MD in patients with isolated recurrent vertigo.

## Materials and Methods

### Subjects

**Figure 1:**
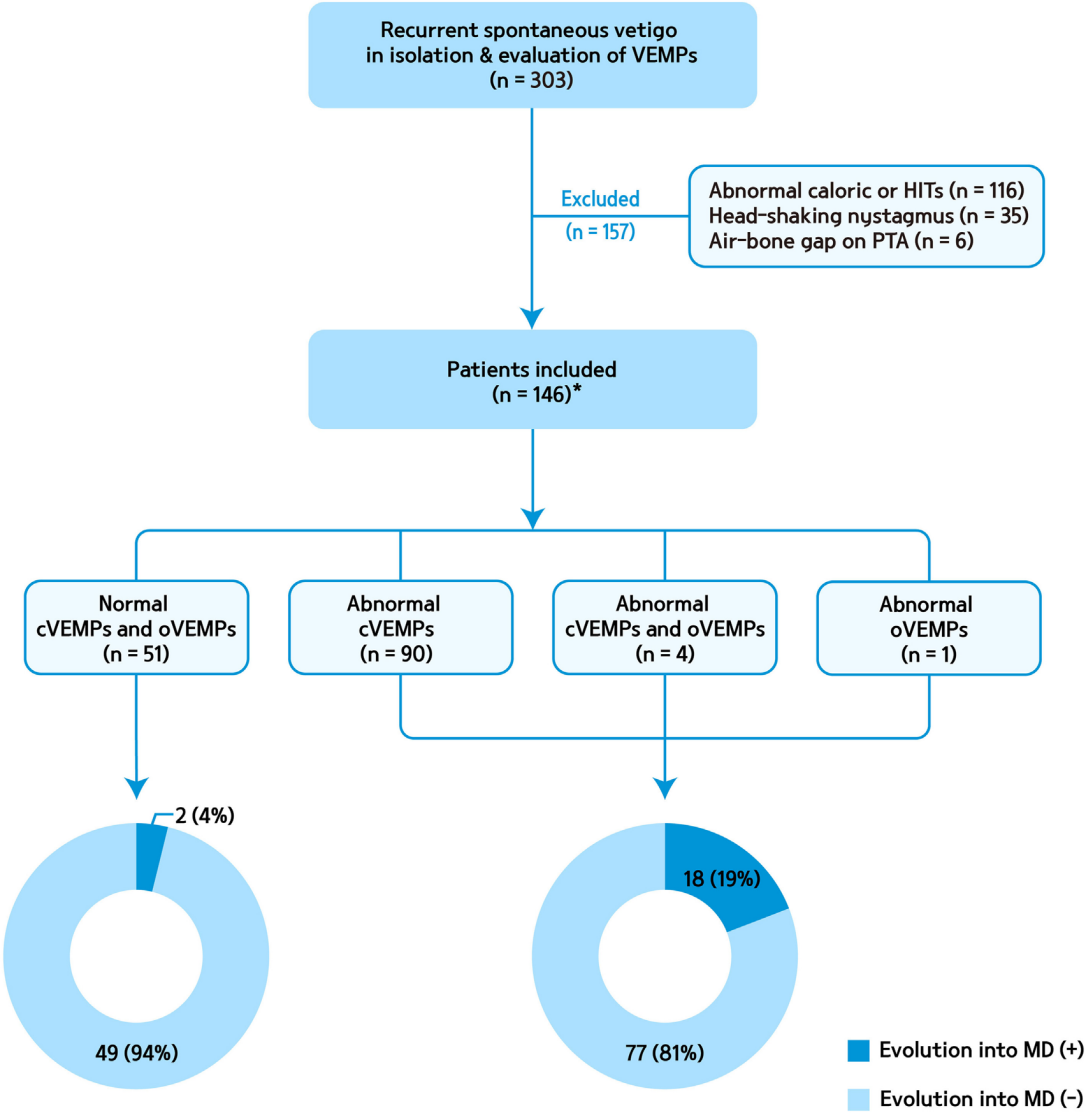
A flow chart for patient selection and evolution into Meniere’s disease (MD) according to abnormalities of cervical and ocular vestibular-evoked myogenic potentials (VEMPs). cVEMP, cervical VEMPs; HITs, head-impulse tests; oVEMP, ocular VEMP; PTA, pure tone audiometry. *oVEMPs were performed in 74 of them.

We retrospectively recruited 303 patients who presented isolated recurrent spontaneous vertigo lasting 20 min to 24 h of unknown causes and had an evaluation of cVEMPs at the Dizziness Clinic of Seoul National University Bundang Hospital from June 2003 to May 2014 (1). oVEMPs were performed in 102 of them. We only included the patients who (1) had more than two attacks of spontaneous rotational vertigo that did not occur during head movements or positional changes, (2) denied migrainous headaches or auditory symptoms during or between the attacks, (3) showed no focal neurologic symptoms or signs, and (4) had no other disorders that may explain the recurrent vertigo. We further excluded those with caloric paresis in either ear (canal paresis >25% or reduced responses in both ears with sum of slow phase velocity of the nystagmus <20°/s), abnormal head-impulse tests (either during bedside or video head-impulse tests, *n* = 116), head-shaking nystagmus (horizontal peak slow phase velocity of nystagmus >2.9°/s or perverted nystagmus, *n* = 35), and air-bone gap on PTA (*n* = 6). Finally, we included 146 patients with isolated idiopathic recurrent vertigo and evaluation of cervical and/or ocular VEMPs (Figure 1). Cervical and/or ocular VEMPs were conducted within 2 months of the inclusion in most patients [117/146, 80%, median = 22 days, interquartile range (IQR) = 9–42 days].

For every patient, a detailed medical history was taken along with a series of vestibular and audiometric tests that included video-oculography, bithermal caloric tests, bedside or video head-impulse tests, cervical and/or ocular VEMPs, and audiometry (6). Evolution into MD was decided when the patients began to meet the diagnostic criteria for probable or definite MD (1). The affected ear was decided with the side of auditory symptoms including hearing loss, tinnitus, and ear fullness. Brain MRIs were obtained in 98 patients (98/146, 67%), which did not show any lesion responsible for the recurrent vertigo.

### Cervical and Ocular VEMPs

Cervical vestibular-evoked myogenic potentials were recorded with the subject supine on a bed with the head raised approximately 30° from the horizontal and rotated contralaterally in order to activate the sternocleidomastoid (SCM) muscles. The surface EMG activity was measured from an active electrode placed over the belly of the contracted SCM after subtracting activity from a reference electrode located on medial clavicle. A ground electrode was attached to the forehead. cVEMPs were recorded using a Nicolet Viking Select unit (Nicolet-Biomedical, Madison, WI, USA). A short burst of alternating tone (110 dB nHL, 123.5 dB SPL, 500 Hz, rise time = 2 ms, plateau = 3 ms, fall time = 2 ms) was applied at 2.1 Hz monaurally *via* a headphone. The analysis time for each stimulus was 50 ms and responses elicited by up to 80 stimuli were averaged for each test. The signal was bandpass filtered at 30–1,500 Hz, and the mean values of at least two trials were obtained from each ear for all participants. During each recording, the amplified EMG activities of the SCM were also monitored and digitized at 1 kHz using an analog-to-digital converter (NI PCI-4461, National Instruments, Austin, TX, USA). The LabVIEW program (National Instruments) was used to analyze the peak to peak amplitudes and calculate the mean tonic activation during the recording. The absolute cVEMP amplitude was then normalized against the mean tonic activation of the SCM during the recording. To compare the normalized p1 − n1 amplitudes of the cVEMPs between the sides, the interaural difference ratio of the normalized amplitudes (IAD_amp_, %) was also calculated as [(Ar − Al)/(Ar + Al) × 100], where Ar and Al are the normalized p1 − n1 amplitude on the right and left sides, respectively. Both the p1 and n1 peak latencies were also calculated. Normative data of cVEMPs were obtained from 28 age-matched healthy subjects (13 men, mean age = 52.3 ± 10, *p* = 0.073) with no history of auditory or vestibular disorders (7).

Ocular vestibular-evoked myogenic potentials were recorded with the subject sitting while looking at a target that was displaced more than 2 m from the eyes and at an angle of more than 20° upward. EMG activities were recorded using surface electrodes. An active electrode was placed 1 cm below the center of the lower eyelid and the reference electrode was attached to the cheek 2 cm below the active electrode. The ground electrode was located on the forehead at Fpz. oVEMPs were elicited by tapping the hairline at AFz using an electric reflex hammer (VIASYS Healthcare, CA, USA). oVEMPs were recorded using a Nicolet Viking Select unit (NicoletBiomedical). Bilateral responses were simultaneously obtained whenever tapping stimuli were applied. oVEMPs in response up to 60 stimuli were averaged for each test, and the average latency of the initial negative peak (n1) and the n1 − p1 amplitude were analyzed. The interaural difference ratio of the amplitude of the oVEMPs was calculated as IAD_amp_ (%) [(Ar − Al)/(Ar + Al) × 100], where Ar and Al are the n1 − p1 amplitude on the right and left sides, respectively (7).

### Pure Tone Audiometry

All patients underwent PTA using air- and bone-conducted signals in an acoustic booth. The hearing threshold was measured at 0.25, 0.5, 1, 2, 3, 4, and 8 kHz. The audiometric evidence for definite MD was defined as low- to medium-frequency sensorineural hearing loss documented with PTA (1). If multiple audiograms were performed before treatment, and demonstrated fluctuation of hearing threshold, the pure tone threshold was decided by the hearing threshold of higher (i.e., worse) one.

### Statistical Analysis

The Kaplan–Meier product-limit method was used to calculate cumulative progression rates. Differences between the groups were analyzed using the log-rank test. Weighted log-rank tests (Peto–Prentice log-rank test; ρ = 1) (8) was applied to minimize the effects of rare incidence of evolution into MD and different follow-up periods among the patients. Cox proportional hazards models were used for univariate and multivariate prognostic analyses. All variables with a *p*-value <0.2 using univariate analysis were included in the multivariate analysis. Then, all variables with a *p*-value <0.05 were decided to be significant in the multivariate analysis. All analyses were performed with R release 4.3.3 software.

This study followed the tenets of the Declaration of Helsinki and obtained an approval from the Institutional Review Board of Seoul National University Bundang Hospital (B-1109/135-106).

## Results

### Evolution of Isolated Recurrent Vertigo into MD

Table 1 summarizes the clinical findings and results of audiometry and VEMPs in the patients. The mean and median durations of the follow-up were 20 (SD = 30) and 6 months (IQR = 0–29).

**Table 1:** Clinical and laboratory findings in the patients with and without evolution into MD.

	Evolution into MD (+) (*n* = 20)	Evolution into MD (−) (*n* = 126)	*p*-Value
Age	53 ± 13	53 ± 14	0.994
Female sex (%)	13/20 (65)	98/126 (78)	0.214
Mean follow-up periods (months)	20 ± 26	17 ± 27	0.648
Attack frequency (/year)	7 ± 7	8 ± 21	0.769
Attack duration (h)	3 ± 3	4 ± 3	0.161
cVEMP abnormalities (%)	18/20 (90)	76/126 (60)	0.011
oVEMP abnormalities (%)	1/10 (10)	4/64 (6)	0.527
**PTA threshold (kHz)**		
0.25	16 ± 14	9 ± 6	0.047
0.50	15 ± 15	10 ± 8	0.223
1	15 ± 15	11 ± 7	0.043
2	16 ± 15	12 ± 9	0.202
3	20 ± 17	16 ± 13	0.270
4	23 ± 20	20 ± 15	0.449
8	35 ± 24	30 ± 20	0.398

*cVEMPs, cervical vestibular-evoked myogenic potentials; MD, Meniere’s disease; oVEMPs, ocular VEMPs; PTA, pure tone audiometry*.

Of the 146 patients finally included in this study, 20 showed an evolution into MD (14%, eight with definite and 12 with probable MD, 18 with unilateral and two with bilateral MD) by developing cochlear symptoms later. The initial cochlear symptoms included tinnitus (*n* = 14), ear fullness (*n* = 9), and hearing loss (*n* = 5). The interval between the onset of vertigo and the development of cochlear symptoms ranged from 1 month to 13.6 years (median = 3 years, IQR = 0.5–4.5 years).

In patients with an evolution into MD (*n* = 20), the median interval from the onset of follow-up to conversion was 7 months (IQR range = 2–33). The cumulative progression rate was 12% [95% confidence interval (CI) = 5–18] at 1 year, 18% (8–26) at 2 years, and 22% (11–32) at 3 years.

### Findings of VEMPs in Isolated Recurrent Vertigo

Cervical vestibular-evoked myogenic potentials were abnormal in 94 (94/146, 65%) patients, which included absent responses in 20 (10 on the left, 5 on the right, and the remaining 5 on both sides), delayed responses in 16 (8 on the left, 8 on the right, and the remaining 3 on both sides) and asymmetric responses in 65. Four patients showed both delayed and asymmetric responses and three patients showed absent responses in one ear, and delayed responses in the other ear.

Of the 94 patients with abnormal cVEMPs, 18 (19%) showed an evolution into MD, while only 2 of the 51 (4%) patients with normal cVEMPs evolved into MD during the follow-up (Figure 1). Overall, in 20 patients with an evolution into MD, cVEMPs were depressed (*n* = 12), augmented (*n* = 5), normal (*n* = 2), absent (*n* = 2, including one with absent responses in one ear, and depressed in the other ear), or delayed (*n* = 1, both delayed and depressed VEMPs in one ear). Among 18 patients with an evolution into unilateral MD, abnormal cVEMP responses were observed during stimulation of the lesioned ear in 16 patients (16/18, 89%), including depressed (*n* = 8), augmented (*n* = 5), absent (*n* = 2), or delayed responses (*n* = 1). cVEMP responses were usually normal during stimulation of the healthy ear (13/18, 72%). However, the remaining five patients showed abnormal cVEMPs which included depressed (*n* = 5) or delayed responses (*n* = 2, both delayed and depressed responses). Two patients with bilateral MD showed decreased responses in both ears. Between the patients with and without an evolution into MD, there were no differences in the p13 n23 latencies, normalized amplitude, and IAD_amp_ (Table 2).

**Table 2:** Cervical and ocular VEMPs in the patients.

		Evolution into MD (+)		Evolution into MD (−)	*p*-Value
		Lesioned ear	Opposite ear		
cVEMPs	p13 (ms)	15.1 ± 1.3	15.1 ± 1.4	15.3 ± 1.5	0.729
	n23 (ms)	23.8 ± 2.1	24.1 ± 1.7	24.2 ± 2.2	0.778
	Normalized amplitude (μV)	2.7 ± 1.9	2.9 ± 1.3	3.5 ± 1.9	0.137
	IAD_amp_ (%)	32 ± 27		27 ± 29	0.430
oVEMPs	n1 (ms)	7.1 ± 0.9	7.1 ± 1.0	6.8 ± 1.2	0.614
	p1 (ms)	11.8 ± 1.5	12.3 ± 2.4	11.4 ± 1.9	0.401
	IAD_amp_ (%)	4.2 ± 4.0		8.5 ± 6.7	0.071

*VEMPs, vestibular-evoked myogenic potentials; cVEMPs, cervical vestibular-evoked myogenic potentials; IAD_amp_, interaural difference of the normalized amplitudes; MD, Meniere’s disease; oVEMPs, ocular VEMPs*.

Among the 74 patients with a testing of oVEMPs, four showed unilateral (two during right ear stimulation, and the other two during left ear stimulation), and one showed bilateral abnormalities. Among the five patients with abnormal oVEMPs, one with bilaterally delayed responses developed unilateral MD during the follow-up while the other four remained free of cochlear symptoms. There were no differences in the n1, p1 latencies, and IAD_amp_ between those with and without an evolution into MD (Table 2).

### Relationship between VEMP Abnormalities and Evolution into MD

**Figure 2:**
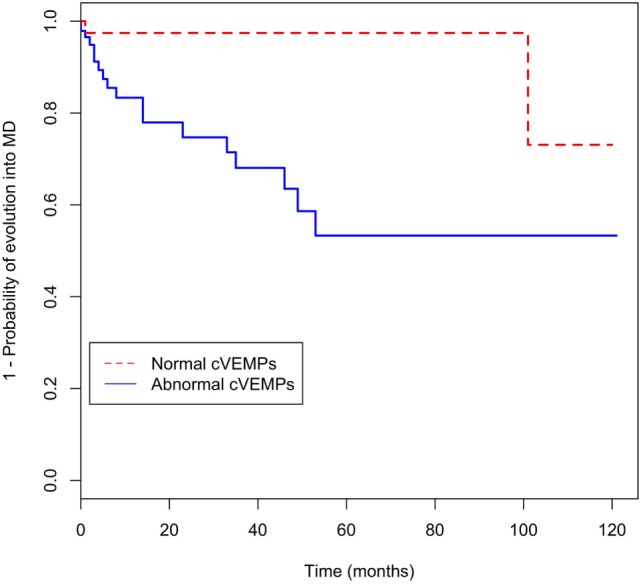
Kaplan–Meier curve for evolution of recurrent vertigo into Meniere’s disease (MD) according to abnormalities of cervical vestibular-evoked myogenic potentials (cVEMPs).

In univariate analysis, progression was associated with PTA threshold at 0.25, 0.5 kHz, and abnormal cVEMPs (Table 3). In addition, PTA threshold at 0.25 kHz [hazard ratio (HR) = 1.1, 95% CI = 1.0–1.2] and abnormalities of cVEMPs (HR = 5.6, 95% CI = 1.3–25.5) were found to be significantly associated with a later conversion into MD with multivariate analysis (Table 3). Patients with abnormal cVEMPs showed a more frequent evolution into MD than those with normal cVEMPs (*p* = 0.01, Figure 2).

**Table 3:** Univariate and multivariate prognostic analyses for predicting MD conversion.

Variables	Univariate analysis		Multivariate analysis	
	HR (95% CI)	*p*-value	HR (95% CI)	*p*-Value
Age	1.00 (0.97–1.03)	0.933		
Female sex	1.95 (0.78–4.9)	0.155	0.75 (0.28–1.99)	0.563
Attack frequency(/year)	1.00 (0.98–1.03)	0.753	1.00 (0.97–1.03)	0.920
Abnormal cVEMPs	5.59 (1.28–24.35)	0.022*	5.64 (1.25–25.50)	0.025*
Abnormal oVEMPs	1.98 (0.24–16.27)	0.523		
PTA threshold at 0.25 kHz	1.05 (1.02–1.08)	0.002*	1.09 (1.00–1.19)	0.041*
PTA threshold at 0.50 kHz	1.04 (1.00–1.07)	0.031*	0.97 (0.89–1.05)	0.429
PTA threshold at 1 kHz	1.04 (1.00–1.07)	0.054	0.97 (0.90–1.05)	0.487
Timing of evaluation since vertigo spell	0.99 (0.98–1.00)	0.118	0.99 (0.98–1.00)	0.177

*cVEMPs, cervical vestibular-evoked myogenic potentials; HR, hazard ratio; CI, confidence interval; oVEMPs, ocular VEMPs; PTA, pure tone audiometry*.

*p < 0.05.

## Discussion

Our study showed that the PTA threshold at 0.25 kHz and abnormal cVEMPs are significantly associated with a later conversion into MD in patients with recurrent spontaneous vertigo in isolation.

Previously, Slater et al. introduced a group of patients who presents recurrent spells of spontaneous vertigo without any auditory symptoms (9). The term “benign recurrent vertigo (BRV)” was coined to describe this disorder that shares common features with vestibular migraine or MD. Indeed, a genetic link has been raised between BRV and MD with familial studies (10, 11). Besides, one-fourth of the relatives of the patients with MD experience spontaneous episodes of vertigo without auditory symptoms (10). Indeed, the term vestibular MD has been adopted in the earlier diagnostic criteria for MD to embrace those patients who develop auditory symptoms later (4). However, existence of this variance was questioned later since patients with vestibular MD showed no clear evidence of EH involved in the disease process. Furthermore, vestibular migraine can be an alternative explanation for the vertigo episodes or a comorbid condition in those patients (12). As the relationship between BRV and vestibular MD has not been clearly elucidated (13), we aimed to determine the role VEMP abnormality as a biomarker for predicting an evolution of isolated recurrent vertigo into MD. Our hypothesis was that associated otolithic dysfunction is an indicator for further progression of isolated recurrent vertigo into MD. Indeed, a combination of cVEMPs and distortion product otoacoustic emissions (OAEs) permits early diagnosis of MD by showing improvements after glycerol administration (14). Our study also implicates that electrophysiologic changes can precede the clinical symptoms and signs of MD. Indeed, VEMPs may predict whether vestibular symptoms will occur to meet the diagnostic criteria of MD in patients with hearing loss (15). Results of VEMPs also correlate well with histopathologic changes of the saccule (15).

Although cVEMPs had a role in predicting evolution of BRV into MD, oVEMPs did not. In other words, the results of cervical and ocular VEMPs were mostly dissociated in our patients with BRV that had evolved into MD. The EH is known to involve the vestibular organs in an orderly manner, mostly starting from the cochlear apex and then to saccule, utricle, and ampullae (16). Indeed, the EH prevails in the cochlea, followed by the saccule that is the vestibular organ closest to the cochlea (5). Due to this adjacency, the cochlear and saccular cells appear to be similarly affected by mechanical bulging of EH from the earliest stage. Histopathologic studies of the temporal bones of MD patients also support this idea; similar dilation of the cochlear duct and saccule is observed, whereas dilation of the utricle and canals is less frequent (5). Otherwise, the resistance to volume increment of an endolymph-filled closed system would be different according to its geometric configuration and distensibility of the membrane of each vestibular structure (17). The walls of the superior labyrinth (utricle and semicircular canals) are thicker than those of the inferior labyrinth (saccule and cochlear), which could explain their relative resistance to mechanical distortion (18, 19). Indeed, a mathematical model showed that the SCCs are most resistant to hydropic expansion while the saccule is most vulnerable (20).

Our patients with an evolution into MD were associated with low-tone hearing impairment on PTA. We may expect that the results of PTA and cVEMPs are correlated since mechanical compression by EH may affect the saccule and cochlea similarly. Indeed, the IAD_amp_ ratio of cVEMPs correlates with the clinical staging of MD which is determined by the hearing level on PTA (21). Since EH starts from the cochlear apex that responds to low-frequency sound stimuli, low-tone sensitivity may be affected initially by EH (22). Although lower tone hearing impairments are associated with progression into MD, absence of cutoff value limits their application to an individual patient. Given the decreased amplitude of OAE even in the unaffected ear of MD patients (23) and its correlation with the stages of MD (24), OAE may also help early diagnosis of MD. However, OAE is limited by its high false-positive rate since the results are largely affected by pathologies involving the external or middle ear (25).

The results of VEMPs in our patients were variable to show absent, depressed, normal, or augmented responses. Indeed, asymmetry of cervical or ocular VEMPs does not necessarily indicate the affected side, especially during the earliest stage of MD (26, 27). This variability may confuse interpretation of VEMP findings in MD (15, 21, 27–29). We believe that the histopathological status of the saccule may determine the VEMP responses (15): If the sensory epithelium in the saccular macula is totally degenerated, the VEMP responses may be absent. Likewise, the dilated saccule with intact saccular macula may delay VEMPs. The dilated saccule with atrophic saccular macula would show depressed VEMPs. Otherwise, the VEMPs may be augmented if the saccular hydrops displaces the extended footplate attachment, thereby making the saccular macula more sensitive to loud sounds to augment VEMPs (30).

Abnormal cVEMPs may also occur in patients with vestibular migraine (31, 32). Since vestibular migraine is also diagnosed in nearly 20% of patients with MD (2) and the life time prevalence of migraine is increased up to 56% in patients with MD (33), the abnormal cVEMPs observed in our patients may be ascribed to comorbid vestibular migraine. However, we excluded the patients who met the diagnostic criteria of vestibular migraine, and none of our patients developed vestibular migraine during the follow-up. Otherwise, abnormal cVEMPs, especially delayed responses, may be observed in central lesions. However, none of our patients showed any focal neurologic deficits on examination, or lesions on brain imaging. In this study, we included only the patients with isolated VEMP abnormalities. This purification of patients may have influenced the results. However, the objective of this study was to determine the role of isolated cVEMP abnormality in predicting future evolution into MD in patients with idiopathic recurrent vertigo.

Of interest, five patients with an evolution into unilateral MD showed abnormal cVEMPs during stimulation of either ear. We suspect that the abnormal cVEMPs in the contralateral ear may reflect asymptomatic EH. Indeed, 15–27% of unilateral MD patients have bilateral VEMP changes (22, 29). In addition, recent advances in electocochleography (34) and intratympanic gadolinium-enhanced MRIs (35) have allowed detection of asymptomatic EH, and predict a progression from unilateral to bilateral MD. The incidence of bilateral diseases ranges from 9 to 50% (36–38). This variability may be ascribed to various diagnostic criteria for bilateral MD and different follow-up periods among the literatures. In this regard, histopathologic studies have found more consistent portion of bilaterality at 25–30% (39–41). Our results indicate that electrophysiologic changes may precede clinical symptoms of MD and abnormalities of cVEMPs are a predictor for evolution of isolated recurrent vertigo into MD.

## Ethics Statement

All experiments followed the tenets of the Declaration of Helsinki and this study was approved by Institutional Review Board of Seoul National University Bundang Hospital (B-1109/135-106).

## Author Contributions

S-UL wrote the manuscript, and analyzed and interpreted the data. H-JK, J-YC, and J-WK analyzed and interpreted the data, and revised the manuscript. J-SK designed and conceptualized the study, interpreted the data, and revised the manuscript.

## Conflict of Interest Statement

S-UL, H-JK, J-YC, and J-WK have no potential conflicts of interest to disclose. J-SK serves as an Associate Editor of *Frontiers in Neuro-otology* and on the Editorial Boards of *Journal of Clinical Neurology, Frontiers in Neuro-ophthalmology, Journal of Neuro-ophthalmology, Journal of Vestibular Research*, and *Journal of Neurology, and Medicine*.
